# Albumin in Sepsis and Septic Shock: A Systematic Review and Meta-Analysis

**DOI:** 10.7759/cureus.108831

**Published:** 2026-05-14

**Authors:** Uday Shree Akkala Shetty, Ashutosh Sharma, Ansharah-E-Zinnia Batool, Helai Hussaini, Sonalben Chaudhary, Rahman Hameed Mohammed Abdul, Calvin R Wei, Shamsha Hirani

**Affiliations:** 1 Internal Medicine, Southern Regional Medical Center, Riverdale, GEO; 2 Internal Medicine, Kathmandu Medical College and Teaching Hospital, Kathmandu, NPL; 3 Medicine, Rashid Latif Medical College, Lahore, PAK; 4 Internal Medicine, West Anaheim Medical Center, Anaheim, USA; 5 Internal Medicine, Zydus Sitapur Hospital, Sitapur, IND; 6 Gastroenterology and Hepatology, Royal Derby Hospital, Stoke-on-Trent, GBR; 7 Research and Development, Shing Huei Group, Taipei, TWN; 8 Cardiology, Baqai Hospital, Karachi, PAK

**Keywords:** albumin, meta-analysis, mortality, sepsis, septic shock

## Abstract

Sepsis and septic shock remain leading causes of mortality in intensive care units (ICUs) worldwide, and the optimal resuscitation fluid continues to be debated. This systematic review and meta-analysis evaluated the effectiveness of human albumin solution compared with control fluids across four patient-centred outcomes in adults with sepsis or septic shock. A comprehensive search of MEDLINE, Embase, Cochrane Central Register of Controlled Trials (CENTRAL), and Cumulative Index to Nursing and Allied Health Literature (CINAHL) was conducted from inception to March 2026. Both randomised controlled trials (RCTs) and observational studies were eligible for inclusion. Nine studies were included in this meta-analysis. Pooled analysis using random-effects models demonstrated that albumin administration was not associated with a statistically significant reduction in all-cause mortality (risk ratio (RR) 1.05, 95% confidence interval (CI) 0.86-1.28), renal replacement therapy (RRT) requirement (RR 1.23, 95% CI 0.70-2.15), or acute kidney injury (AKI) incidence (RR 1.09, 95% CI 0.85-1.40). The sole outcome demonstrating consistent and statistically significant benefit was cumulative fluid balance, which was significantly lower in albumin-treated patients (mean difference (MD) -0.79 L, 95% CI -1.16 to -0.41). Subgroup analysis suggested that the renal effect of albumin is concentration-dependent, with hyperoncotic preparations associated with greater RRT risk in patients with pre-existing renal impairment. Albumin use is associated with a lower short-term cumulative fluid balance with no additional benefits on RRT and AKI. However, adequately powered randomised trials stratified by albumin concentration are needed to provide definitive guidance.

## Introduction and background

Sepsis and septic shock represent life-threatening emergencies characterised by a dysregulated host response to infection, resulting in organ dysfunction and haemodynamic instability [1]. Globally, sepsis affects more than 48 million individuals annually, contributing to approximately 11 million deaths, making it one of the leading causes of mortality in intensive care units (ICUs) worldwide [2]. Fluid resuscitation remains a cornerstone of early sepsis management, aimed at restoring intravascular volume, maintaining tissue perfusion, and preventing progressive organ dysfunction [3]. Current international guidelines recommend crystalloid solutions as the primary resuscitation fluid, with human albumin solution proposed as an adjunct when large volumes of crystalloid are required [4].

Human albumin is a naturally occurring colloid with multiple physiological properties beyond its oncotic effects, including antioxidant activity, nitric oxide scavenging, modulation of capillary permeability, and carrier functions for endogenous and exogenous compounds [5]. These pleiotropic properties have provided a compelling biological rationale for its use in critically ill patients with sepsis, where systemic inflammation, capillary leak, and profound hypoalbuminaemia are common [6]. Hypoalbuminaemia itself is independently associated with increased mortality, prolonged ICU stay, and greater organ dysfunction in septic patients, further supporting the theoretical basis for albumin supplementation [7].

Despite this rationale, clinical evidence regarding the efficacy of albumin in sepsis remains inconsistent. The landmark Saline versus Albumin Fluid Evaluation (SAFE) study demonstrated no overall mortality difference between albumin and saline in a heterogeneous ICU population, though a predefined subgroup analysis suggested a potential survival benefit in patients with severe sepsis [8]. Subsequently, the Albumin Italian Outcome Sepsis (ALBIOS) trial, which randomised 1,818 patients with severe sepsis to 20% albumin targeting a serum level of 30 g/L or crystalloid alone, reported no improvement in 28-day or 90-day mortality, despite demonstrable haemodynamic advantages [9]. More recently, the ARISS trial similarly found no significant reduction in 90-day mortality with albumin replacement in septic shock, though the trial was prematurely terminated due to low enrolment [10].

Beyond mortality, the effects of albumin on clinically important outcomes, including acute kidney injury (AKI), renal replacement therapy (RRT) requirement, and cumulative fluid balance, remain poorly defined, with existing trials underpowered to detect differences in these secondary endpoints [11]. Furthermore, emerging evidence suggests that the concentration of albumin administered - iso-oncotic (4-5%) versus hyperoncotic (20-25%) - may differentially influence renal outcomes, with hyperoncotic preparations potentially exerting detrimental effects on glomerular filtration in patients with pre-existing renal impairment [12].

Given these uncertainties, an updated systematic review and meta-analysis incorporating both randomised controlled trials (RCTs) and observational studies is warranted to comprehensively evaluate the effectiveness of albumin across the full spectrum of patient-centred outcomes in sepsis and septic shock. This review, therefore, aimed to determine the effect of albumin administration on mortality, AKI, RRT requirement, ICU length of stay, and cumulative fluid balance in adult patients with sepsis or septic shock.

## Review

Methodology

This systematic review and meta-analysis was conducted and reported in accordance with the Preferred Reporting Items for Systematic Reviews and Meta-Analyses (PRISMA) 2020 guidelines [13].

Eligibility Criteria

Studies were eligible for inclusion if they met the following predefined criteria. The population of interest comprised adult patients (aged ≥18 years) with a confirmed or suspected diagnosis of sepsis or septic shock, as defined by the criteria used in each individual study, including the 1992 American College of Chest Physicians/Society of Critical Care Medicine (ACCP/SCCM) consensus definitions or the Third International Consensus Definitions for Sepsis and Septic Shock (Sepsis-3). Table 1 presents the inclusion and exclusion criteria for study selection.

**Table 1:** Eligibility criteria for selection of studies

Category	Criterion
Inclusion criteria	
Population	Adult patients aged ≥18 years with a confirmed or suspected diagnosis of sepsis or septic shock, as defined by the 1992 ACCP/SCCM consensus definitions or the Third International Consensus Definitions for Sepsis and Septic Shock (Sepsis-3).
Intervention	Administration of human albumin solution of any concentration (4%, 5%, 20%, or 25%) via any route and at any dose.
Comparator	Any control fluid including crystalloids, normal saline, balanced crystalloids, or no albumin.
Outcomes	Studies reporting at least one of the prespecified outcomes: all-cause mortality, renal replacement therapy requirement, acute kidney injury incidence or cumulative fluid balance.
Study design	Randomised controlled trials (RCTs) or observational studies.
Language	Studies published in the English language. We included conference abstract if data comparing two treatments available.
Exclusion criteria	
Population	Studies conducted exclusively in paediatric populations. Studies conducted in animal models. Studies conducted in animal models.
Data	Studies reporting insufficient data for quantitative synthesis.
Study type	Reviews, meta-analysis, editorials, case studies, and case series.

Outcomes

The primary outcome was all-cause mortality, reported at the longest available follow-up time point (28-day, 30-day, or 90-day mortality). Secondary outcomes included (1) requirement for RRT; (2) incidence of AKI, defined according to any validated criteria, including Risk, Injury, Failure, Loss of Kidney Function, and End-Stage Kidney Disease (RIFLE), Kidney Disease: Improving Global Outcomes (KDIGO), or National Institute for Health and Care Excellence (NICE) guidelines; and (3) cumulative fluid balance.

Information Sources and Search Strategy

A comprehensive and systematic literature search was conducted across four electronic databases: MEDLINE (via PubMed), Embase, the Cochrane Central Register of Controlled Trials (CENTRAL), and the Cumulative Index to Nursing and Allied Health Literature (CINAHL). The search was conducted from database inception to 20 March 2026 without language or date restrictions. The search strategy incorporated Medical Subject Headings (MeSH) terms and free-text keywords relating to the population (sepsis, septic shock, severe sepsis), intervention (albumin, human albumin solution, colloid), and study design (randomised controlled trial, cohort study, observational study). Boolean operators (AND, OR) were used to combine search terms. In addition to database searching, the reference lists of all included studies and relevant systematic reviews were manually screened to identify any additional eligible studies not captured by the electronic search. Trial registries, including ClinicalTrials.gov and the WHO International Clinical Trials Registry Platform (ICTRP), were also searched to identify ongoing or unpublished trials. Table 2 presents the search strategy for each database used.

**Table 2:** Search strategy

Database	Search strategy
MEDLINE (via PubMed)	#1 "sepsis"[MeSH Terms] OR "septic shock"[MeSH Terms] OR "severe sepsis"[tiab] OR "sepsis"[tiab] OR "septic shock"[tiab]#2 "albumin"[MeSH Terms] OR "serum albumin"[MeSH Terms] OR "human albumin solution"[tiab] OR "albumin"[tiab] OR "colloid"[tiab] OR "hyperoncotic albumin"[tiab] OR "iso-oncotic albumin"[tiab]#3 "randomized controlled trial"[pt] OR "randomised controlled trial"[tiab] OR "cohort study"[tiab] OR "observational study"[tiab] OR "retrospective study"[tiab] OR "propensity score"[tiab]#4 #1 AND #2 AND #3
Embase	#1 'sepsis'/exp OR 'septic shock'/exp OR 'severe sepsis':ti,ab OR 'sepsis':ti,ab OR 'septic shock':ti,ab#2 'albumin'/exp OR 'human albumin'/exp OR 'human albumin solution':ti,ab OR 'albumin':ti,ab OR 'colloid':ti,ab OR 'hyperoncotic albumin':ti,ab OR 'iso-oncotic albumin':ti,ab#3 'randomized controlled trial'/exp OR 'randomised controlled trial':ti,ab OR 'cohort analysis'/exp OR 'observational study':ti,ab OR 'retrospective study':ti,ab OR 'propensity score':ti,ab#4 #1 AND #2 AND #3
Cochrane Central Register of Controlled Trials (CENTRAL) (Cochrane)	#1 MeSH descriptor: [Sepsis] explode all trees OR MeSH descriptor: [Shock, Septic] explode all trees OR "severe sepsis":ti,ab OR "sepsis":ti,ab OR "septic shock":ti,ab#2 MeSH descriptor: [Albumins] explode all trees OR "human albumin solution":ti,ab OR "albumin":ti,ab OR "colloid":ti,ab OR "hyperoncotic albumin":ti,ab OR "iso-oncotic albumin":ti,ab#3 #1 AND #2Date range:
Cumulative Index to Nursing and Allied Health Literature (CINAHL)	#1 (MH "Sepsis+") OR (MH "Shock, Septic") OR TI ( "sepsis" OR "septic shock" OR "severe sepsis" ) OR AB ( "sepsis" OR "septic shock" OR "severe sepsis" )#2 (MH "Albumins+") OR TI ( "albumin" OR "human albumin solution" OR "colloid" OR "hyperoncotic albumin" OR "iso-oncotic albumin" ) OR AB ( "albumin" OR "human albumin solution" OR "colloid" )#3 (MH "Randomized Controlled Trials") OR (MH "Cohort Studies+") OR TI ( "randomised controlled trial" OR "cohort study" OR "observational study" OR "propensity score" ) OR AB ( "randomised controlled trial" OR "cohort study" OR "observational study" OR "propensity score" )#4 #1 AND #2 AND #3

Study Selection

All records identified through the electronic database search were imported into Rayyan (Rayyan Systems Inc., Cambridge, MA) systematic review software, where duplicate records were automatically identified and removed. Two independent reviewers screened all titles and abstracts against the predefined eligibility criteria. Full-text articles were subsequently retrieved for all records deemed potentially eligible at the title and abstract screening stage. Two reviewers independently assessed full-text articles for eligibility, and any disagreements were resolved through discussion and, where necessary, by consultation with a third reviewer. The reasons for exclusion of full-text articles were documented and are reported in the PRISMA flow diagram.

Data Extraction

A standardised data extraction form was developed and piloted prior to use. Two reviewers independently extracted data from each included study, with discrepancies resolved by consensus. The following data were extracted from each eligible study: first author and year of publication; study design; country and setting; sample size; albumin concentration, comparator fluid, duration of follow-up; baseline patient characteristics, including age and sex, and proportion with septic shock; and outcome data for all prespecified outcomes. For continuous outcomes, means and standard deviations (SDs) were extracted where available. Where studies reported medians and interquartile ranges (IQRs) rather than means and SDs, values were converted to means and SDs using the method described by Wan et al. [14].

Assessment of Methodological Quality and Risk of Bias

The methodological quality of included RCTs was assessed independently by two reviewers using the Cochrane Risk of Bias tool version 2 (RoB 2), which evaluated bias arising from the randomisation process, deviations from intended interventions, missing outcome data, measurement of the outcome, and selection of the reported result [15]. Observational studies were assessed using the Newcastle-Ottawa Scale (NOS), which evaluates studies across three domains: selection of study groups, comparability of groups, and ascertainment of the outcome of interest [16]. Studies were categorised as being at low, moderate, or high risk of bias based on the overall assessment. Disagreements between reviewers were resolved through discussion or adjudication by a third reviewer.

Statistical Analysis

All statistical analyses were performed using R software (version 4.3.0; R Foundation for Statistical Computing, Vienna, Austria) with the meta and metafor packages. For dichotomous outcomes, including mortality, RRT requirement, and AKI incidence, pooled effect estimates were calculated as risk ratios (RRs) with 95% confidence intervals (CIs). For continuous outcomes, including cumulative fluid balance, pooled mean differences (MDs) with 95% CIs were calculated.

Given the anticipated clinical and methodological heterogeneity across included studies, random-effects models were applied for all pooled analyses as the primary approach. Statistical heterogeneity was quantified using the I² statistic and Cochran's Q test, with I² values of 25%, 50%, and 75% considered to represent low, moderate, and substantial heterogeneity, respectively.

Prespecified subgroup analyses were conducted to explore potential sources of heterogeneity and to assess whether treatment effects differed according to: (1) albumin concentration (iso-oncotic 4-5% versus hyperoncotic 20-25%). Sensitivity analyses were performed using a leave-one-out approach, whereby each study was systematically omitted from the pooled analysis to assess its influence on the overall result and on heterogeneity estimates. A two-sided p-value of less than 0.05 was considered statistically significant for all analyses.

Results

**Figure 1:**
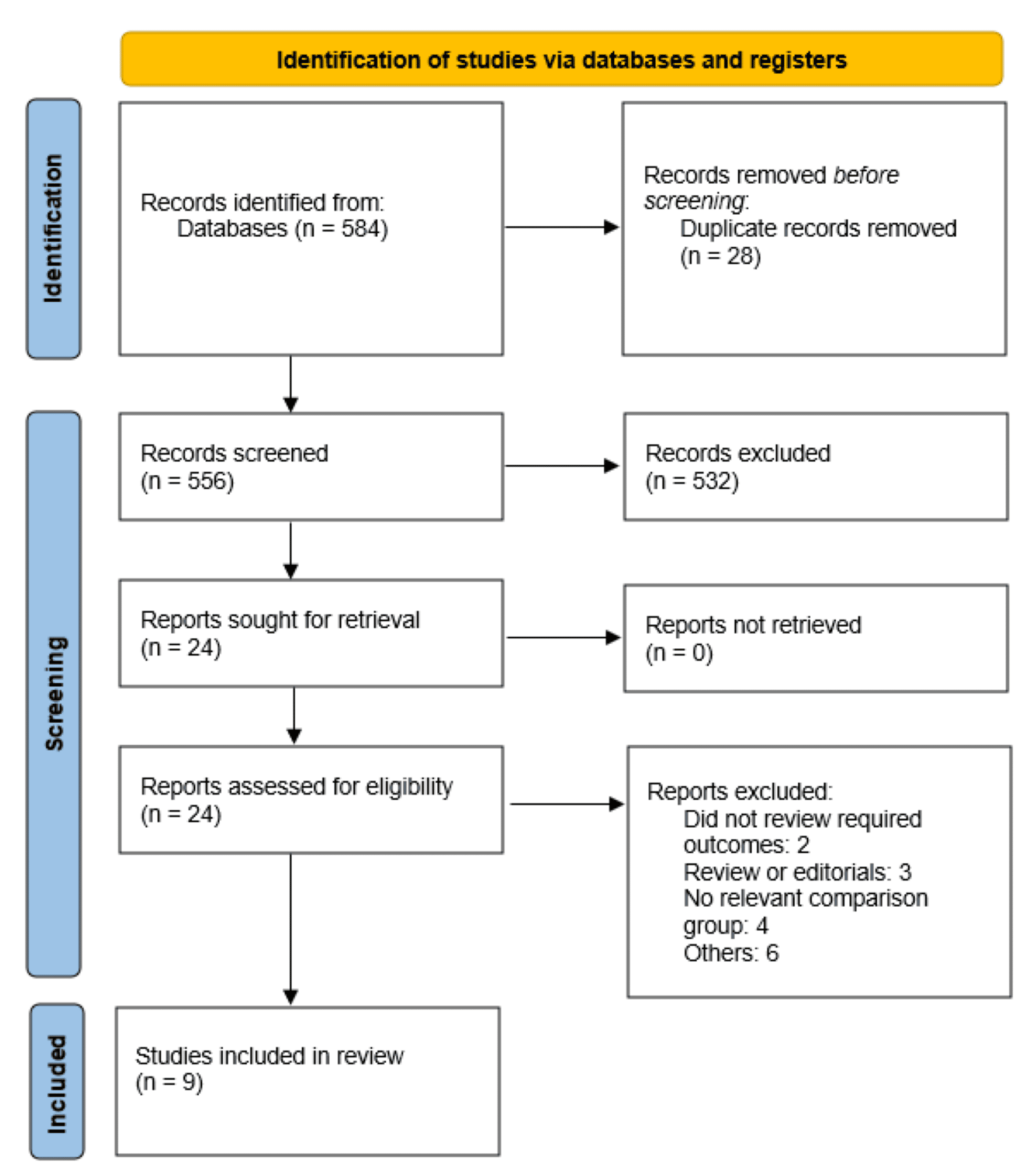
Study selection process (PRISMA)

Through online database searching, we identified 584 studies. Duplicate records were removed, followed by an initial screening of titles and abstracts. Full-text records of 24 studies were retrieved and assessed for eligibility. After detailed evaluation based on predefined inclusion and exclusion criteria, nine studies were included in the final meta-analysis. Figure 1 illustrates the PRISMA flowchart of the study selection process, and Table 3 summarises the characteristics of the included studies.

**Table 3:** Characteristics of included studies ICU - intensive care unit; NHS - National Health Service (UK); NR - not reported; RCT - randomised controlled trial

Author, year	Design	Setting/region	Sample size (n)	Follow-up	Albumin: concentration and dose	Comparison group	Age (years)	Males, n (%)	Baseline serum albumin
Caironi et al., 2014 [9]	RCT - multicentre, 100 ICUs	Italy (ICU)	Total: 1,810. Albumin: 903. Comparison: 907.	90 days	Concentration: 20%	Crystalloid alone	Albumin: 70. Comparison: 69.	Albumin: 543. Comparison: 550.	Albumin: 24.1 g/L. Comparison: 24.2 g/L.
Charpentier et al., 2011 [17]	RCT	USA	Total: 792. Albumin: 399. Comparison: 393.	28 days	Concentration: 20%	0.9% saline	NR	NR	NR
Dolecek et al., 2009 [18]	RCT	USA	Total: 46. Albumin: 26. Comparison: 20.	28 days	Concentration: 20%	250ml 6% starch	Albumin: 44. Comparison:44.	NR	NR
Gray et al., 2024 [19]	RCT - multicentre, 15 NHS hospitals	United Kingdom (emergency departments)	Total: 300. Albumin: 147. Comparison: 149.	90 days	Concentration: 5%	Balanced crystalloid	Albumin: 70. Comparison: 69.	Albumin: 71. Comparison: 80.	NR
Liu et al., 2025 [20]	Observational	Taiwan	Total: 376,730. Albumin: 188,365. Comparison: 188,365.	30 days	Not specified	Standard care	Albumin: 67.8. Comparison: 67.6.	Albumin: 99,107. Comparison:99,750.	NR
Maiwall et al., 2022 [21]	RCT - single-centre, (emergency department + liver ICU)	India (single centre)	Total: 100. Albumin: 50. Comparison: 50.	28 days	Concentration: 20%	Balanced crystalloid	Albumin: 50.6. Comparison 47.3.	Albumin: 44. Comparison: 44.	Albumin: 2.13 g/dL. Comparison: 2.26 g/dL.
Patanwala et al., 2025 [12]	Observational	USA	Total: 9,988. Comparison: 7,929. Albumin: 2,059.	In-hospital	Concentration: 5% or 25%	No albumin during hospitalisation	Comparison: 68.4. Albumin: 65.6.	NR	NR
The SAFE Study Investigators, 2011 [8]	RCT - multicentre, 16 ICUs	Australia and New Zealand (ICU)	Total: 1,218. Albumin: 603. Control: 615.	28 days	Concentration: 4%	0.9% normal saline	Albumin: 60.5. Comparison: 61.0.	Albumin: 359. Comparison: 351.	Albumin: 25.0 g/L. Comparison: 25.2 g/L.
Sakr et al., 2026 [10]	RCT - multicentre, 23 ICUs	Germany (ICU)	Total: 440. Albumin: 222. Comparison: 218.	90 days	Concentration: 20%	Crystalloid alone	Albumin: 70. Comparison 69.	Albumin: 142. Comparison: 148.	Albumin: 2.2 g/dL. Comparison: 2.2 g/dL.

The included studies comprised a mix of RCTs and observational studies conducted across diverse geographical regions, including Italy, the United States, the United Kingdom, Taiwan, India, Australia, New Zealand, and Germany. Most studies were conducted in ICU settings, with some involving emergency departments and specialised units such as liver ICUs. Sample sizes varied widely, ranging from small single-centre trials with fewer than 50 participants to large-scale observational cohorts exceeding 300,000 patients.

The duration of follow-up differed across studies, with most reporting outcomes at 28 or 90 days, while some assessed in-hospital or 30-day outcomes. Albumin administration protocols also varied, with concentrations ranging from 4% to 25%, and comparisons commonly made against crystalloid solutions, including 0.9% saline, balanced crystalloids, or starch-based fluids, as well as standard care without albumin. Table 4 and Table 5 present the quality assessment of the included studies.

**Table 4:** Quality assessment of included studies using Risk of Bias tool version 2 (RoB 2)

Study	Randomisation	Deviations from intended interventions	Missing outcome data	Outcome measurement	Selection of reported result	Overall judgement
Caironi et al., 2014 [9]	Low	Some concerns	Low	Low	Low	Some concerns
Charpentier et al., 2011 [17]	Low	Low	Low	Low	Low	Low
Dolecek et al., 2009 [18]	Low	Low	Low	Low	Some concerns	Some concerns
Gray et al., 2024 [19]	Low	Some concerns	Low	Low	Low	Some concerns
Maiwall et al., 2022 [21]	Low	High	Low	Low	Low	High
The SAFE Study Investigators [8]	Low	Low	Low	Low	Some concerns	Some concerns
Sakr et al., 2026 [10]	Low	Some concerns	Low	Low	Low	Some concerns

**Table 5:** Quality assessment of included observational studies using Newcastle-Ottawa Scale (NOS)

Study	Selection	Comparability	Outcome	Overall
Liu et al., 2025 [20]	3	2	2	High
Patanwala et al., 2025 [12]	4	2	2	High

Mortality

**Figure 2:**
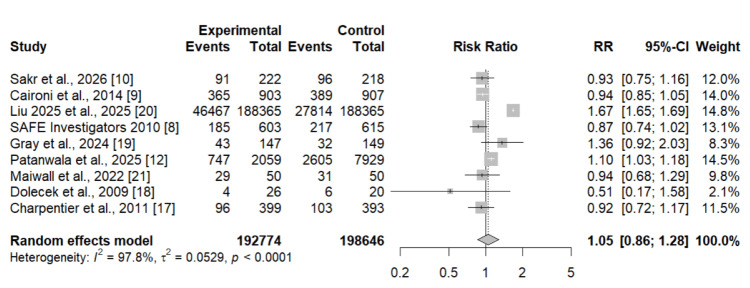
Forest plot of mortality Experimental group: albumin; control group: comparison References: [8-10,12,17-21]

A total of nine studies comprising 391,420 patients (192,774 in the albumin group and 198,646 in the control group) were included in the mortality meta-analysis, and the results are shown in Figure 2. Using a random-effects model, albumin administration was not associated with a statistically significant difference in mortality compared with control treatment (RR = 1.05, 95% CI: 0.86-1.28). There was substantial heterogeneity among the included studies (I² = 97.8%, p < 0.001). Individual study estimates showed variability in effect direction; however, the pooled estimate crossed the line of no effect, indicating no overall mortality benefit or harm associated with albumin therapy.

Sensitivity Analysis of Mortality

**Figure 3:**
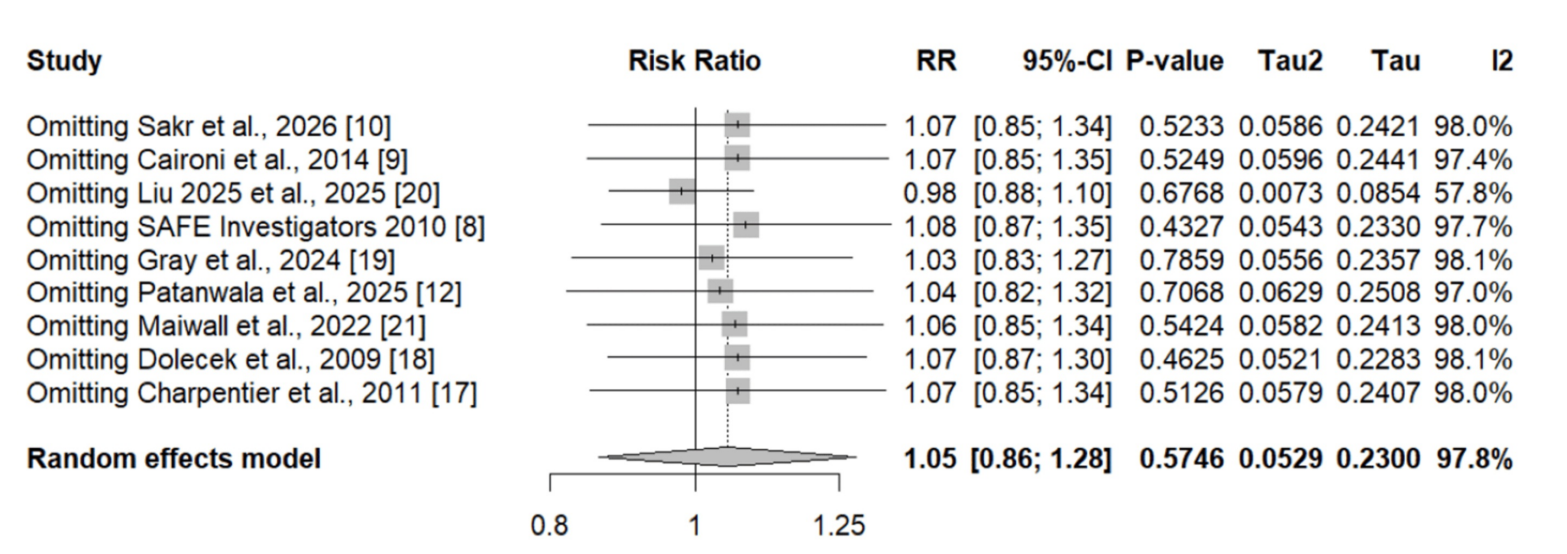
Sensitivity analysis of mortality

Leave-one-out sensitivity analysis was performed to evaluate the influence of individual studies on the pooled mortality estimate, and the results are presented in Figure 3. Sequential exclusion of each study did not materially alter the overall effect size, with pooled RRs ranging from 0.98 (95% CI: 0.88-1.10) after omission of the study by Liu et al. [20] to 1.08 (95% CI: 0.87-1.35) after omission of the SAFE Investigators [8]. The overall pooled estimate remained non-significant across all iterations, indicating that no single study disproportionately influenced the results. The mortality RR of 1.67 in Liu et al. [20] is an outlier compared to all other studies; this study was observational, and confounding by indication is likely: sicker patients received albumin, inflating the harm signal. Additionally, dose, concentration, timing, and duration are entirely unknown, making it a fundamentally different "intervention" compared to the protocol-driven RCTs.

RRT

**Figure 4:**
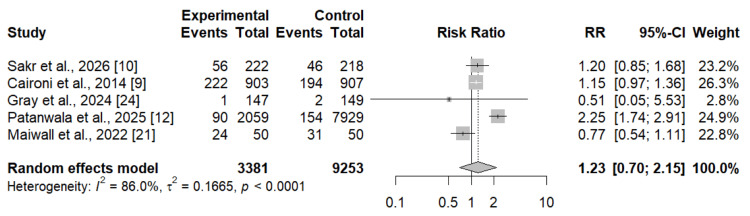
Forest plot (RRT) Experimental group: albumin; control group: comparison References: [9,10,12,19,21]

Five studies involving 12,634 patients (albumin: n = 3,381; control: n = 9,253) reported data on RRT use. Pooled analysis using a random-effects model demonstrated no statistically significant difference in RRT requirement between albumin and control groups (RR 1.23, 95% CI 0.70-2.15), although the point estimate favoured the control group as shown in Figure 4. Substantial heterogeneity was detected across studies (I² = 86.0%, τ² = 0.1665, p < 0.0001), precluding definitive conclusions from the pooled estimate.

Cumulative Fluid Balance

**Figure 5:**
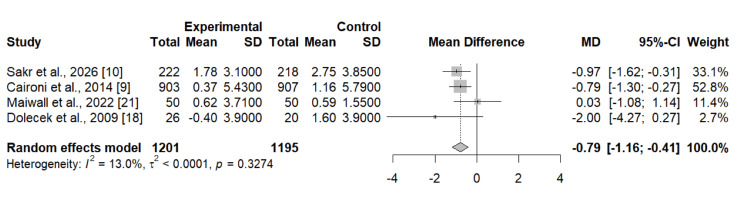
Forest plot (cumulative fluid balance) Experimental group: albumin; control group: comparison References: [9,10,18,21]

Four studies were included to compare the cumulative fluid balance between the albumin and control groups, and the results are shown in Figure 5. Pooled analysis showed that cumulative fluid balance was significantly lower in subjects receiving albumin compared to the control group (MD: -0.79, 95% CI: -1.16 to -0.41). Low heterogeneity was reported among the study results (I² = 13%).

AKI

**Figure 6:**
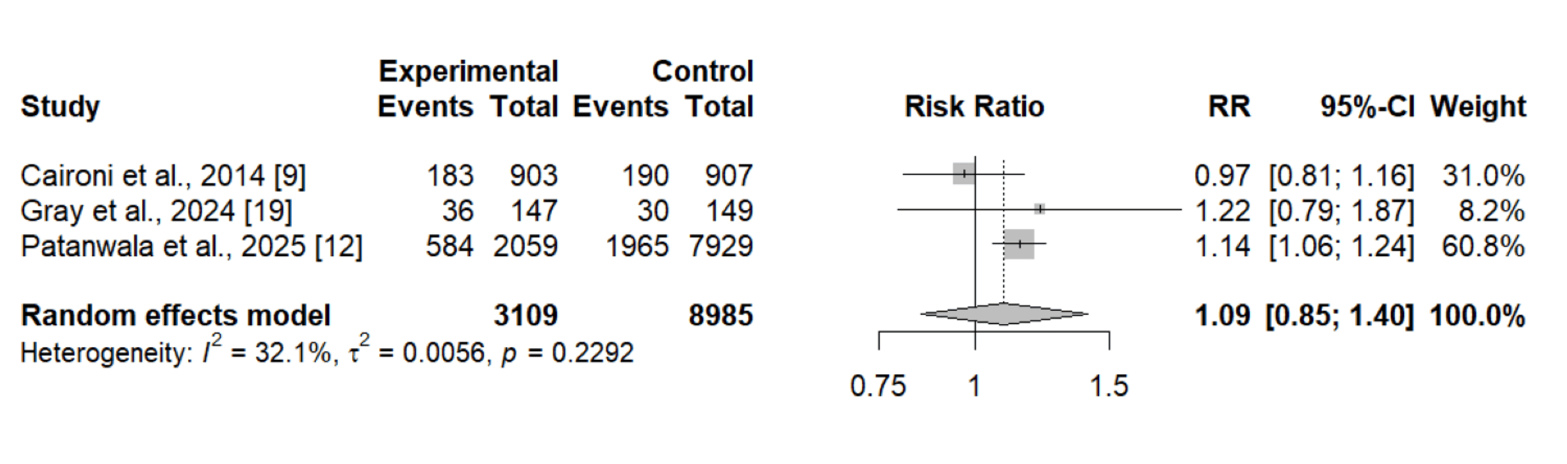
Forest plot (AKI) Experimental group: albumin; control group: comparison References: [9,12,19]

Three studies compared the risk of AKI between the albumin and control groups, and the results are shown in Figure 6. Pooled analysis showed that the risk of AKI was not significantly different between the two groups (RR: 1.09, 95% CI: 0.85 to 1.40). No significant heterogeneity was reported among the study results.

Subgroup Analysis

Table 6 presents the subgroup analysis based on the type of albumin solution used (hypertonic vs. isotonic). For mortality, the risk appeared lower with hypertonic solutions, although the difference was not statistically significant, whereas isotonic solutions were associated with a slightly higher risk, also without statistical significance. Regarding RRT, albumin use showed an effect, but only one study evaluated isotonic solutions, so these findings should be interpreted with caution.

**Table 6:** Subgroup analysis CI: confidence interval; RRT: renal replacement therapy; RR: risk ratio

Outcomes	Subgroups	RR (95% CI)	I²
Mortality	Hypertonic	0.93 (0.85 to 1.01)	51.50%
	Isotonic	1.05 (0.68 to 1.62)	20.70%
RRT	Hypertonic	1.04 (0.83 to 1.30)	32.30%
	Isotonic	0.51 (0.05 to 5.53)	NA

Discussion

This systematic review and meta-analysis evaluated the effectiveness of albumin solution across four prespecified patient-centred outcomes - mortality, RRT requirement, AKI, and cumulative fluid balance - in adults with sepsis or septic shock. The principal finding was that albumin administration, compared with control fluids, was not associated with a statistically significant reduction in all-cause mortality, RRT requirement, or AKI incidence. However, albumin was associated with a significantly lower cumulative fluid balance, suggesting a clinically meaningful fluid-sparing effect. Taken together, these findings are consistent with the existing body of evidence and reinforce the view that albumin cannot be recommended as a routine resuscitation fluid across all patients with sepsis or septic shock, though specific subpopulations and clinical contexts may derive benefit [22].

The absence of a statistically significant mortality benefit with albumin in this meta-analysis is consistent with findings from the two largest RCTs to date. The ALBIOS trial, which enrolled 1,818 patients with severe sepsis, demonstrated no survival advantage with 20% albumin at 28 or 90 days despite demonstrable haemodynamic improvements, including higher mean arterial pressure and a more negative fluid balance [9]. Similarly, the ARISS trial, the only RCT with 90-day mortality as its primary endpoint, found no significant reduction in 90-day mortality in patients with septic shock (RR 0.94, 95% CI 0.76-1.17), though its premature termination - enrolling only 440 of the intended 1,662 patients - rendered the trial substantially underpowered [10]. Furthermore, a large meta-analysis by Patel et al. utilising trial sequential analysis similarly concluded that existing evidence was insufficient to confirm or refute a mortality benefit of albumin in sepsis, highlighting the persistent need for adequately powered trials [23].

The very high heterogeneity in the mortality analysis (I² = 97.8%) was largely driven by Liu et al. [20], whose omission reduced I² to 57.8% with a shift in pooled RR from 1.03 to 0.97. This observational database study is susceptible to confounding by indication, as albumin was identified by diagnostic codes without data on dose, concentration, or timing, and propensity score matching cannot fully account for unmeasured severity variables [20,24]. Its disproportionate sample size (n = 376,730) further amplified its influence on the pooled estimate. These findings support restricting primary meta-analyses to RCTs, with observational studies included only as sensitivity analyses [25].

The pooled RRT analysis showed no significant overall difference, though the effect was markedly concentration-dependent. Pathophysiologically, albumin maintains oncotic pressure and attenuates sepsis-induced renal vasoconstriction through nitric oxide-scavenging and anti-inflammatory properties [5,6]; however, hyperoncotic preparations may paradoxically increase intraglomerular oncotic pressure and reduce glomerular filtration, as previously demonstrated with high-molecular-weight colloids [26]. Consistent with this, Patanwala et al. reported significantly increased RRT risk with 25% albumin (OR 1.43, 95% CI 1.16-1.76) but not with 5% albumin (OR 1.07, 95% CI 0.84-1.37) in patients with pre-existing renal impairment [12]. Conversely, the Albumin in Liver Disease and Sepsis-induced Hypotension (ALPS) trial demonstrated a renal-protective effect of 20% albumin in cirrhotic patients (RRT 38% vs. 62%, p = 0.03), consistent with its established role in attenuating hepatorenal syndrome - confirmed by Sort et al. in spontaneous bacterial peritonitis [21,27]. The effect of albumin on RRT is therefore both concentration-dependent and population-specific.

AKI incidence was not significantly different between groups. Given AKI affects up to two-thirds of patients with septic shock and is associated with a six- to eight-fold increase in hospital mortality [28], the null finding likely reflects inadequate power and heterogeneous AKI definitions across studies rather than a true absence of effect.

The most consistent benefit was a significantly lower cumulative fluid balance with albumin (MD -0.79 L, 95% CI -1.16 to -0.41; I² = 13%), reflecting its oncotic efficiency in drawing interstitial fluid intravascularly and achieving haemodynamic targets with less administered volume [5]. The Small Volume Resuscitation with 20% Albumin in Intensive Care: Physiological Effects (SWIPE) trial demonstrated this most clearly (300 mL vs. 900 mL for equivalent haemodynamic response) [29], and ALBIOS confirmed a significantly lower net fluid balance (347 mL vs. 1,220 mL, p = 0.004) [9]. Since positive fluid balance independently predicts prolonged mechanical ventilation, ICU stay, and mortality [30, 31], this fluid-sparing effect may represent albumin's most consistently realisable clinical benefit.

Comparison With Existing Systematic Reviews

The findings of this review are broadly concordant with prior systematic reviews and meta-analyses, which have consistently failed to demonstrate a statistically significant mortality benefit for albumin in patients with sepsis or septic shock [11]. Delaney et al. reported a trend toward reduced mortality with albumin in patients with sepsis, but this did not reach statistical significance [11]. A meta-analysis by Geng et al. [31] specifically evaluated different albumin concentrations in patients with sepsis and septic shock and identified a significant mortality reduction with 20% albumin compared with crystalloids, driven largely by data from the ALBIOS septic shock subgroup, while no benefit was demonstrated with 4-5% albumin [32]. An earlier meta-analysis by Wilkes and Navickis [31] demonstrated that albumin-treated patients had improved survival compared to controls, with a pooled relative risk of 0.82 (95% CI 0.67-1.00), suggesting a possible mortality benefit that subsequent larger trials have been unable to confirm definitively [33]. More recently, a Cochrane review by Roberts et al. confirmed the absence of a clear mortality benefit for albumin across critically ill populations while noting the specific biological plausibility of benefit in sepsis-related hypoalbuminaemia [25]. The present review extends prior work by incorporating more recent high-quality studies, including the ARISS trial in 2026 and the large observational analyses by Liu et al. [20] and Patanwala et al. [12], and by comprehensively evaluating RRT, AKI, and fluid balance as co-primary outcomes alongside mortality. The inclusion of data on cumulative fluid balance, an outcome largely overlooked in prior meta-analyses, represents a novel contribution and highlights a consistent benefit of albumin that may have practical implications for clinical management.

Current international guidelines offer a nuanced and conditional endorsement of albumin in sepsis management. The Surviving Sepsis Campaign (SSC) 2021 guidelines recommend the use of crystalloids as the primary resuscitation fluid and make a weak recommendation - based on moderate-quality evidence - to use albumin in patients who have received substantial volumes of crystalloid and continue to require vasopressors [3,4]. Albumin is not recommended as a replacement for crystalloids during initial resuscitation. The European Society of Intensive Care Medicine (ESICM) 2024 fluid therapy guidelines provide a conditional recommendation favouring crystalloids over albumin as a resuscitation fluid in critically ill patients with sepsis, citing the lack of definitive mortality benefit alongside the substantially higher cost of albumin preparations.

Strengths and Limitations

This review has several strengths. It is the most up-to-date systematic review and meta-analysis on this topic, incorporating the recently published ARISS trial in 2026, which was the first RCT specifically designed to investigate 90-day mortality as a primary endpoint in septic shock. The review evaluated multiple clinically relevant outcomes beyond mortality, including AKI, RRT, and fluid balance, providing a more complete picture of the risk-benefit profile of albumin. Prespecified subgroup and sensitivity analyses were conducted to explore sources of heterogeneity and the influence of individual studies. The inclusion of both RCTs and observational studies maximised data availability for secondary outcomes that were frequently unreported in individual trials.

Several limitations must be acknowledged. First, very high heterogeneity was observed in the primary mortality analysis (I² = 97.6%), driven principally by the inclusion of large observational database studies with fundamentally different methodological approaches, populations, and exposure definitions compared to the RCTs. Second, the studies included in this review differed substantially in albumin concentration (4-25%), dose, administration protocol, target serum albumin level, and duration of therapy, as well as in the comparator used and the definition of sepsis applied. These differences limit the validity of pooled estimates and may obscure genuine concentration-specific or population-specific effects. Third, outcomes were measured at heterogeneous timepoints across studies, which was particularly problematic for cumulative fluid balance analysis, where measurements ranged from 48 hours to 28 days. Fourth, the ALPS trial enrolled exclusively cirrhotic patients, a population with distinct haemodynamic physiology, and inclusion of this study in the broader sepsis meta-analysis may introduce population-level confounding. Fifth, data on fluid balance were sparse, with only four studies contributing to this analysis, and the two largest trials contributing median and IQR data that required conversion to means and SDs using established statistical methods, introducing additional uncertainty. Finally, publication bias cannot be fully excluded, particularly for smaller studies reporting favourable outcomes with albumin, though the limited number of studies precluded formal funnel plot analysis for most outcomes.

Implications for Clinical Practice and Future Research

The current evidence does not support the routine use of albumin as a primary resuscitation fluid in unselected patients with sepsis or septic shock, and this review reinforces the recommendation of the SSC that crystalloids should remain the first-line fluid [3]. However, the data also do not support a blanket avoidance of albumin. In specific clinical contexts - including patients with profound hypoalbuminaemia, patients with cirrhosis and sepsis-induced hypotension, and patients in whom positive fluid balance is a significant concern - albumin may offer meaningful advantages, particularly in terms of fluid balance and haemodynamic stability. The consistent fluid-sparing effect of albumin, especially hyperoncotic 20% albumin, suggests that it may have a role as a volume-efficient resuscitation adjunct to reduce the burden of fluid accumulation and its downstream consequences in selected high-risk patients. In cirrhotic patients with sepsis-induced hypotension specifically, the haemodynamic rationale for albumin use extends beyond oncotic pressure to include its anti-inflammatory and nitric oxide-scavenging properties, which may directly attenuate the vasodilatory state characteristic of advanced liver disease complicated by infection [31].

The use of hyperoncotic albumin in patients with pre-existing renal impairment warrants particular caution, given the concentration-dependent renal risk identified by Patanwala et al. [12]. Clinicians should be cognisant of the potential for 25% albumin (the North American formulation equivalent to European 20% albumin) to increase RRT requirement in this population, and iso-oncotic preparations may be preferable where albumin administration is clinically indicated in patients with renal compromise [12]. Future research should prioritise large, adequately powered RCTs that pre-specify albumin concentration as a stratification variable, recruit patients based on contemporary Sepsis-3 definitions, and evaluate patient-centred outcomes, including RRT requirement, fluid balance, and health-related quality of life, alongside mortality. The ongoing focus on whether non-oxidised or functionally active albumin may yield superior outcomes compared to standard commercial preparations also represents an important avenue for translational investigation [10].

## Conclusions

This systematic review and meta-analysis of nine studies demonstrates that albumin administration in patients with sepsis or septic shock does not significantly reduce all-cause mortality, RRT requirement, or AKI incidence compared with control fluids. However, albumin was associated with a clinically and statistically significant reduction in cumulative fluid balance, a finding that is mechanistically consistent and may have important implications for the prevention of fluid overload and its consequences in critically ill patients. The high degree of heterogeneity across studies, driven largely by methodological differences between RCTs and large observational database studies, together with variation in albumin concentration, patient population, and clinical context, precludes definitive conclusions about the overall effectiveness of albumin. The evidence suggests a concentration-dependent effect, with hyperoncotic albumin potentially associated with greater renal risk in patients with pre-existing renal impairment, while its fluid-sparing properties may be advantageous in selected patients. Routine albumin supplementation cannot be recommended for all patients with sepsis or septic shock based on current evidence. Future well-designed, adequately powered RCTs stratified by albumin concentration and incorporating comprehensive renal and fluid balance endpoints are necessary to provide definitive guidance on the role of albumin in the management of this critically ill population.
